# Predictors of COVID-19 Vaccine Intention: Evidence from Chile, Mexico, and Colombia

**DOI:** 10.3390/vaccines10071129

**Published:** 2022-07-15

**Authors:** Camila Salazar-Fernández, María José Baeza-Rivera, Marcoantonio Villanueva, Joaquín Alberto Padilla Bautista, Regina M. Navarro, Mariana Pino

**Affiliations:** 1 Departamento de Análisis de Datos, Universidad Autónoma de Chile, Temuco 4813302, Chile; camila.salazar@uautonoma.cl; 2 Laboratorio de Interacciones, Cultura y Salud, Departamento de Psicología, Facultad de Ciencias de la Salud, Universidad Católica de Temuco, Temuco 4810101, Chile; 3 Programa de Doctorado en Psicología, Universidad de La Frontera, Temuco 4811230, Chile; mvillanueva02@ufromail.cl; 4 Facultad de Ingeniería y Negocios Guadalupe Victoria, Universidad Autónoma de Baja California, Mexicali 21289, Mexico; joaquin.padilla@uabc.edu.mx; 5 CEPEC, Centro de Excelencia en Psicología Económica, Universidad de La Frontera, Temuco 4811230, Chile; reginanb@gmail.com; 6 Programa de Psicología, Universidad Autónoma del Caribe, Barranquilla 080020, Colombia; mariana.pino@gmail.com

**Keywords:** vaccine hesitancy, conspiracy theories, social influence, vaccination behavior, vaccine behavior, coronavirus

## Abstract

(1) Background: Although the evidence is consistent that vaccines for COVID-19 effectively prevent severe illness or death, the rapid development of vaccines has led to increased beliefs about possible negative consequences and conspiracy theories about the vaccine. Several factors influence whether or not people decide to be vaccinated. Some studies suggest that our perception of what significant others do and think influences our behavior. (2) Methods: This study evaluates the predictive role of beliefs about negative consequences of the COVID-19 vaccine, conspiracy beliefs about this vaccine, and social influence on the intention to vaccinate against COVID-19 in three Latin American and Caribbean countries: Chile, Mexico, and Colombia. Using convenience sampling, 2075 adults from Chile (48.3%), Mexico (27.6%), and Colombia (24.6%) participated by answering an online questionnaire with variables of interest. (3) Results: Despite the differences between countries, the results showed that the proposed model is invariant and explains between 56–66% of the COVID-19 vaccination intent. Specifically, controlling for age, socioeconomic status, political orientation, and educational level, we found that beliefs about the negative consequences of the COVID-19 vaccine were the main predictor followed by social influence. Beliefs in conspiracy theories did not predict vaccination intention (4) Conclusions: Considering these variables in campaigns to boost vaccination intention is discussed.

## 1. Introduction

Since the World Health Organization (WHO) declared the COVID-19 pandemic in March 2020, countries have developed health strategies to control its spread and mitigate its consequences at the public health level [1]. To date, 219 million cases and 4.55 million deaths have been reported worldwide due to the pandemic [2]. To prevent the spread of the virus, the WHO has suggested care measures such as the use of masks, physical distancing, and frequent hand washing, among others [3,4,5,6,7]. However, each country has adopted these measures to its national reality, considering its health system and economic resources. That said, the repercussions of this pandemic have been uneven among countries, with those with fewer resources and weaker health systems being the most affected, for example, those belonging to Latin America and the Caribbean [8].

Historically, vaccines have shown their effectiveness in controlling infectious diseases and viruses [9]. However, evidence in recent years indicates that the acceptance and intention of vaccination have diminished due to an increased mistrust and concerns about the safety of vaccines [10]. In this regard, in 2019 the WHO considered indecision about vaccination as one of the ten main threats to global health [11], representing a severe threat to control of the current COVID-19 pandemic [12].

Several reasons affect people’s confidence in vaccines and their consequent intention to vaccinate. Among these reasons are the perception of vaccines’ safety and efficacy, their potential risks, the compatibility of getting vaccinated with religious beliefs, the lack of information about side effects, and distrust in the medical system [13,14,15]. These negative beliefs about vaccines have increased in recent months [16]. The scenario of uncertainty and the rapid development of vaccines for COVID-19 have caused people to develop negative beliefs about the possible consequences that the vaccine may have (e.g., an eventual increase in the spread of the virus, distrust of its effectiveness in the long term, an increased likelihood of infection and higher risks associated with side effects) [15,17].

The current pandemic has facilitated the configuration of these beliefs into conspiracy theories. Conspiracy theories emerge as a way to satisfy psychological needs such as certainty, understanding, and desire for control and security [18]. Satisfying these needs provides us an answer, an explanation that makes sense to us in a moment when nothing does, due to mistrust in periods of crisis [18,19]. Furthermore, conspiracy beliefs dictate what to believe and whom to trust, modeling the attitudes and behaviors of those who adhere to them [18,20]. Among the emerging conspiracy theories associated with COVID-19 vaccines, beliefs about the virus being a biological weapon developed to control and destabilize the population [21], and that vaccines contain a chip that will allow Bill Gates to have control over people are the ones that stand out [22]. Literature has suggested that believing in conspiracy theories is associated with negative attitudes towards vaccines, and therefore, with less vaccination intention and behavior [23,24,25,26,27,28,29,30,31]. 

Social influence (e.g., social norm) is a variable that has played an essential role in understanding and predicting the performance of health behaviors [32]. In the current pandemic, social influence has shown an association with carrying out prevention behaviors [33,34] and with greater vaccination intention [23]. Social influence refers to our perception of what attitudes or behaviors are approved by significant others, allowing us to meet the reference group’s expectations [35]. In this way, if our reference group executes a behavior, for example, receiving the vaccine, it is more likely that the person has a greater intention of carrying out that behavior (getting vaccinated). Accordingly, the literature on the social amplification of risk suggests that the recognition, interpretation, and communication from others about the potential hazards determine the risk perception of the event and the subsequent behavior to be performed [36,37]. Thus, significant others exert pressure to mobilize an individual’s behavior and safeguard their sense of group belonging [38]. Consequently, social influence plays an important role in vaccination intention [23,39,40]. 

Vaccine intention and vaccination are health behaviors that have been studied by several models, such as the health belief model [41,42,43], the integrative model for the study of culture and health behaviors [44,45,46], or the theory of reasoned action [47]. These models consider psychological and cultural variables (beliefs and social influence) as antecedents of health behaviors. Moreover, sociostructural variables have been conceived as sources of individual variation that can impact health behaviors through psychological and cultural variables [44,45,46]. Specifically, recent studies have shown that sociostructural variables such as low educational level, right-wing political orientation, lower social position, and older age are related to negative beliefs about vaccines, conspiracy theories, and less intention to vaccinate (van Mulukom et al., 2020).

In the present research, we evaluate the intention to vaccinate against COVID-19 in a Chilean, Mexican, and Colombian sample, drawing on the adaptation of the theory of reasoned action for the study of vaccination intention developed by Baeza-Rivera and Salazar-Fernández [23]. Particularly, in this study, we used negative beliefs towards the consequences of the COVID-19 vaccine, conspiracy theories about the COVID-19 vaccine, and the social influence on the vaccination intention for COVID-19 as predictors of vaccination intention.

Specifically, these three countries have different conditions to face the pandemic. For example, in 2020, Chile allocated 5.9% of its GDP to health, while Mexico only allocated 2.5% and Colombia 5.3% [48]. Although every country has followed the guidelines proposed by the WHO to avoid contagion, these have been adapted to the national reality (see Table 1 for contextual information about COVID-19 and the strategies adopted by each country). To achieve the study’s objective, first, the invariance of the model will be tested in each of the nations. Then the predictive role that these variables have in the vaccination intention for COVID-19 will be examined, controlling the effect of sociostructural variables such as educational level, political orientation, social status, and age.

## 2. Materials and Methods

### 2.1. Participants

A total of 1002 people from Chile (48.3%), 563 from Mexico (27.1%), and 510 from Colombia (24.6%) participated in a nonprobability national online survey. The inclusion criterion was that the participants were of legal age (over 18 years old) and that they were currently living in their country during the pandemic. The age range was from 18 to 89 years (*M* = 35.12, *SD* = 14.59) and 69.7% were women. The sociodemographic characteristics of the participants of each country are shown in Supplementary Table S1. Participants from Chile, Mexico, and Colombia display differences according to their age, marital status, socioeconomic status, educational level, and political orientation.

### 2.2. Instruments

The participants answered an online survey that contained different scales about COVID-19 and sociodemographic variables:

#### 2.2.1. Beliefs about Negative Consequences of COVID-19 Vaccine

This instrument by Salazar-Fernández et al. (2021) includes six items that assess beliefs about the negative consequences of a COVID-19 vaccine. To answer, the participants had to indicate, from 1 to 5, their degree of disagreement or agreement with the statements. High scores reflect higher negative beliefs about consequences of a COVID-19 vaccine. This one-dimensional scale reported a good level of reliability (Chile: ω = 0.877, Mexico: ω = 0.818, Colombia: ω = 0.853). 

#### 2.2.2. Conspiracy Beliefs about COVID-19 Vaccine

We used an instrument composed of three items translated and adapted from Brotherton et al. (2013) that evaluate adherence to conspiracy theories regarding COVID-19 vaccine. To answer, the participants had to indicate their degree of disagreement (1) or agreement (5). High scores indicate a greater belief in conspiracy theories about COVID-19 pandemic. This one-dimensional scale reported a good level of reliability (Chile: ω = 0.846, Mexico: ω = 0.799, Colombia: ω = 0.805).

#### 2.2.3. Social Influence on COVID-19 Vaccination Intent

The item “I would consider getting vaccinated against COVID-19 if someone close to me does it” was created to evaluate the effect of the social influence on the intent to vaccinate against COVID-19. To answer, the participants had to indicate, from 1 to 5, their degree of disagreement or agreement with the statement. A high score indicates higher influence of the social norm.

#### 2.2.4. Vaccination Intent against COVID-19 

This item sought to assess the probability of vaccinating against COVID-19. The participants assessed their intent using a scale that ranged from not likely (0) to extremely likely (4). A high score on this item reflected a high probability of vaccination. 

#### 2.2.5. Control Variables 

We included the following sociodemographic characteristics in our model as control variables: socioeconomic status (1 = lower to 6 = higher), political orientation (1 = far-left to 5 = far-right), educational level (1 = no formal education to 8 = postgraduate), and age. Higher scores reflect higher perceived socioeconomic status, subscription to right-wing ideology, higher levels of education, and higher age, respectively.

### 2.3. Data Collection

The ethics committee approved data collection in each of the universities’ countries. The survey was applied using the online platform QuestionPro during the period from December 2020 to April 2021. The link to the survey was disseminated through social networks such as Facebook, Instagram, and WhatsApp. This format allowed access to many participants while reducing the risk of contagion of COVID-19. The survey included informed consent that indicated the study’s objective, ensured anonymity and confidentiality, and provided the contact details of the responsible researchers. Answering the survey took approximately 15 min.

### 2.4. Statistical Analysis

Preliminarily, the data were explored at a descriptive level in each country. Asymmetry and kurtosis were found to be acceptable. The scales’ internal consistency was evaluated using the McDonald omega (Revelle and Zinbarg, 2008). The normality of the variables was corroborated with the Shapiro–Wilk test [49], which allowed us to rule out the normality of the variables. Then, using the *lavaan* package (Rosseel, 2012) of the *R* software (R Core Team, 2020), structural multigroup equation models were estimated using the WLSMV estimation method, which is more suitable for non-normal and ordinal data (Flora and Curran, 2004). Structural equation models allowed us to estimate simultaneous correlations, controlling for the effect of the several variables included in the model. This implies that bivariate associations could disappear when other variables are considered concurrently in the same model. Additionally, this technique has the advantage of modeling the measurement errors [50]. 

Using the proposed method by Milfont and Ficher [51], we tested the proposed model structure on the sample of each country to make sure the model provides a good fit. The estimated models were evaluated according to the following global fit indices: χ^2^, the comparative fit index (CFI), the Tucker Lewis index (TLI), the square root of the standardized mean residuals (SRMR), and the square root of the mean error of approximation (RMSEA) with its confidence interval at 90%. According to the conventional goodness of fit criteria, these indices were interpreted: CFI and TLI > 0.95, and SRMR and RMSEA ≤ 0.08 [52,53].

According to Sass and Schmitt [54], measurement invariance is necessary for testing structural coefficients across groups. Hence, using multigroup structural equation models, we tested a sequence of progressively more restrictive models to prove measurement invariance of our model between countries. If these hierarchical constraints (form, factor loadings, intercepts, and residuals) did not worsen the model fit, we accepted the level of invariance tested (i.e., configural, metric, scalar, and strict, respectively). Once measurement invariance was proved, we decided to evaluate structural invariance, a model in which structural paths (i.e., regression coefficients) are set to equal across samples. Reaching this level of invariance implies that the model works in the same way across countries. To decide whether to accept or reject the invariance model tested, we used the ΔCFI and the ΔRMSEA. According to Sass, Schmitt [55], Rutkowski, and Svetina [56], if ΔCFI < 0.010 and ΔRMSEA < 0.015, we rejected model invariance. 

## 3. Results

Correlations between the mean of items that compose each variable of the present study are shown in Table 2. This analysis revealed moderate and strong associations between predictors and vaccination intent among the Chilean, Mexican, and Colombian data. Also in Table 2 are the mean and standard deviation of the scores of each variable.

### 3.1. Invariance Analysis

Before testing the invariance of the model, we tested the model on each sample separately, without restrictions. The three models showed an excellent fit to the data: Chilean sample—χ^2^ (72) = 138.250, *p* < 0.05, CFI = 0.994, TLI = 0.992, RMSEA = 0.030 [0.023, 0.038], SRMR = 0.036; Mexican sample—χ^2^ (72) = 99.174, *p* < 0.05, CFI = 0.993, TLI = 0.990, RMSEA = 0.026 [0.011, 0.038], SRMR = 0.040; and Colombian sample—χ^2^ (72) = 78.802, *p* < 0.05, CFI = 0.998, TLI = 0.998, RMSEA = 0.014 [0.000, 0.039], SRMR = 0.037. Since the model showed excellent fits for the three samples, in the next step we proceeded to test whether the model was invariant across countries, using multigroup analysis. By imposing sequential restrictions (see M1, M2, M3, and M4 on Table 3) we found that our model showed full strict measurement invariance. Having established strict measurement invariance, we were able to perform meaningful comparisons across groups. For this reason, we tested the structural invariance to explore whether regression coefficients on the model remained invariant (M5). Our analysis revealed that the relationship patterns across the constructs are invariant in the Chilean, Mexican, and Colombian samples. Therefore, our model predicting COVID-19 vaccination intent is equivalent across samples. 

### 3.2. Model Predicting COVID-19 Vaccination Intent

**Figure 1:**
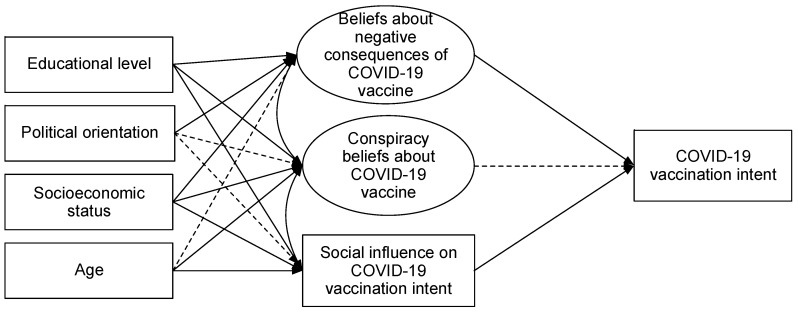
Model (invariant for the Chilean, Mexican, and Colombian sample) predicting COVID-19 vaccination intent. Dashed lines represent nonsignificant paths (*p* > 0.05).

This model (see Figure 1) explained between 56.4% and 66.5% of the variance of COVID-19 vaccination intent. Our data showed negative covariances between beliefs about negative consequences of the COVID-19 vaccine and social influence (*r* = −0.262, *p* < 0.05), and between conspiracy beliefs and social influence (*r* = −0.147, *p* < 0.05). On the contrary, there was a positive covariance between beliefs about negative consequences of the COVID-19 vaccine and conspiracy beliefs about the COVID-19 vaccine (*r* = 0.652, *p* < 0.05). Specifically, we found that when controlling for the sociostructural variables, conspiracy beliefs about the COVID-19 vaccine did not significantly predict COVID-19 vaccination intent (see Table 4). Meanwhile, beliefs about negative consequences of the COVID-19 vaccine and social influence on COVID-19 vaccination intent were significantly associated with COVID-19 vaccination intent. Precisely, our results suggest that people who have beliefs about negative consequences of the COVID-19 vaccine tend to have lower vaccination intent and that those who perceive that close ones would vaccinate report higher vaccination intent for COVID-19.

Regarding the control by sociostructural variables on the predictive variables (see Supplementary Table S2), we found that higher educational level was negatively associated with beliefs about negative consequences of the COVID-19 vaccine (β = −0.151, *p* < 0.05) and with conspiracy beliefs about the COVID-19 vaccine (β = −0.158, *p* < 0.05), and positively related to social influence on COVID-19 vaccination intent (β = 0.072, *p* < 0.05). In the case of political orientation, we did not find a significant association with beliefs about negative consequences of the COVID-19 vaccine or with social influence on COVID-19 vaccination intent (β = 0.041, *p* > 0.05 and β = −0.033, *p* > 0.05, respectively), but we did find a small yet positive association with conspiracy beliefs about the COVID-19 vaccine (β = 0.061, *p* < 0.05). In relation to socioeconomic status, we found a negative association with beliefs about negative consequences of the COVID-19 vaccine and with conspiracy beliefs about the COVID-19 vaccine (β = −0.119, *p* < 0.05 and β = −0.158, *p* < 0.05, respectively), and a positive association with social influence on COVID-19 vaccination intent (β = 0.100, *p* < 0.05). Finally, concerning age we found a null association with beliefs about negative consequences of the COVID-19 vaccine (β = 0.006, *p* > 0.05), a positive association with conspiracy beliefs about the COVID-19 vaccine (β = 0.118, *p* < 0.05) and a negative association with social influence on COVID-19 vaccination intent (β = −0.104, *p* < 0.05).

## 4. Discussion

This study aimed to evaluate the predictive role of beliefs about negative consequences of the COVID-19 vaccine, conspiracy beliefs about the COVID-19 vaccine, and social influence on the vaccination intention for COVID-19 in Colombia, Mexico, and Chile. After establishing the strict invariance of the model which ensured that the model worked equivalently across countries, we tested our model, controlling for sociodemographic variables. Our model shows that negative beliefs about the COVID-19 vaccine and social influence were statistically significant predictors of vaccination intention. In contrast, conspiracy beliefs about the COVID-19 vaccine were not a significant predictor. This model broadens the understanding of the intention to vaccinate against COVID-19 within the context of Latin America and the Caribbean and provides evidence that allows for the establishment of critical variables in the intention to vaccinate against COVID-19.

The results of this study provide evidence that beliefs about the negative consequences of the COVID-19 vaccine are one of the main predictive variables of the intention to vaccinate against COVID-19 ($\bar{\beta }$ = 0.58). This finding is consistent with Rhodes and Hoq [10], who point out that mistrust about the safety of vaccines reduces their acceptance. That said, negative beliefs about the COVID-19 vaccine referring to a possibility of an increase of contracting the virus or more complex effects than the virus itself may be the result of the spreading of false information regarding the vaccine [57], as well as conspiracy theories associated with the pandemic [24,27,30]. These findings align with the positive and large association found between negative beliefs towards the vaccine and conspiracy theories towards the vaccine. Consequently, negative beliefs about the vaccine are a barrier to vaccination intention [21,22,58]. In the present study, we assessed negative beliefs about the COVID-19 vaccine, but future research should also consider positive beliefs. As Garcia and Vargas [15] state, beliefs such as vaccines being a good option or that vaccines help save people could have a vital role in predicting COVID-19 vaccination intent. 

Contrary to other studies in Western contexts, which found an association between conspiracy theories and vaccination intention [24,29,30], we found no evidence for this claim in the present study. However, these results are consistent with those of Baeza-Rivera and Salazar-Fernández [23], which could shed light on the existence of specific characteristics shared in Latin contexts that could be associated to beliefs in conspiracy theories regarding the vaccine. In this context, the literature has suggested that crises, such as the one generated by the current COVID-19 pandemic, promote the emergence of conspiracy theories [18,29]. Nevertheless, unlike other Western and politically stable countries, the Latin American and Caribbean situation is characterized by scenarios of constant political crises and high levels of distrust towards the authorities [59]. That said, a new crisis such as the one generated by the COVID-19 pandemic presumably does not have enough impact for people to guide their behavior according to conspiracy beliefs, or perhaps, these questions may be influenced by social desirability. Future studies should explore whether conspiracy theories act or are modified by the influence of third variables (mediators and/or moderators) such as the political climate and distrust in the authorities.

Regarding social influence, in this study, we found a positive and significant relationship with vaccination intent ($\bar{\beta }$ = 0.261). This positive association is consistent with the work of Cookson and Jolley [39], Sinclair and Agerström [40], Biddlestone and Green [60], and Campos-Mercade and Meier [61], who highlighted the critical role that social influence has had in the context of the COVID-19 pandemic on the development and maintenance of preventive health behaviors, such as vaccination behavior. These findings are also coherent with the social amplification of risk literature because others have a role in determining what risk is and how to act accordingly—in this case, through vaccination. Thus, those who have shown greater concern for collective norms and the self (collectivism), prioritizing the sense of social responsibility, have shown greater adherence to different healthcare behaviors to avoid COVID-19 infection [62]. These findings become relevant in Latin American countries, in which “significant others” such as family and friends have a transcendental role in decision making and behaviors (e.g., familism, a characteristic of Latin and Asian cultures that involves the prioritization of the family over the self; see Schwartz and Weisskirch [63]). Consequently, to increase the intention to vaccinate in collectivist countries, communication strategies should not only promote the positive consequences that vaccines have [64], but rather, vaccination behavior should be promoted as a way to care for and protect those who belong to the nuclear and extended circle [65,66]; that is, emphasizing it as prosocial behavior [67].

The sociodemographic variables used as controls in this study showed different patterns of relationships with the predictive variables of vaccination intention. In this regard, both social status and educational level were negatively related to beliefs about the negative consequences of the COVID-19 vaccine and adherence to conspiracy theories. This finding is consistent with evidence showing that those with higher economic resources and educational levels, because they have greater access to reliable information, adhere less to conspiratorial and negative beliefs about the vaccine [68]. Moreover, political orientation and age did not show significant associations with negative beliefs towards the COVID-19 vaccine but with conspiracy theories. These results are consistent with the evidence that indicates that right-wing and older groups are more susceptible to believing in conspiracy theories [69,70,71].

Despite the robust results reported in this study, some challenges remain that must be resolved in future research. The first is that the data are cross-sectional, which prevents establishing a causal predictive pattern. A longitudinal study would effectively evaluate potential causal relationships and actual vaccination behavior (and not only its intention). Procedural aspects give the second challenge. Due to the COVID-19 pandemic, the data-collection process was carried out online, which inevitably left people who did not have Internet access out of the study. Future studies should consider other data-collection strategies that allow access to all segments of the population. Finally, it is essential to consider that this study was carried out prior to the beginning of the mass-vaccination process in the three countries, so the results presented here reflect only this time period. Future studies should evaluate whether this model is not only invariant between countries, but also over time.

## 5. Conclusions

The present study’s findings allow a comprehensive understanding of the intention to vaccinate in Latin America and the Caribbean. More importantly, despite the differences between the countries at the macroindicator level (i.e., population, percentage of the GPD dedicated to health), their sociodemographics, and how each country has managed the pandemic, the model proposed by Baeza-Rivera et al. (2021) works maintaining the same relationship structure between the variables. The proposed model constitutes an opportunity to develop joint strategies aimed at promoting vaccination intent and behavior, as well as other health behaviors that seek to control the pandemic in Latin countries. These strategies should focus on vaccination campaigns that provide the necessary information to dispel myths and false beliefs about the vaccine and its effects in the short, medium, and long term [72], and promote vaccination behavior as a way of collective care [60,61,67].

## Figures and Tables

**Table 1:** Contextual information about COVID-19 and COVID-19 vaccination in Chile, Mexico, and Colombia.

	Chile	Mexico	Colombia
Population (2021)	19,107,000	128,970,000	51,450,738
Date of data collection	December 2020 to January 2021	January to April 2021	February to April 2021
COVID-19 cases (Accumulated to the time of data collection)	727,109	2,344,755	2.859,724
COVID-19 deaths (Accumulated to the time of data collection)	18,452	216,907	73,720
Start of mass vaccination process for COVID-19	3 February 2021	16 February 2021	17 February 2021
Vaccinated for COVID-19 to November 2021	83.8%	50.1%	47.3%
Ranking in the comparison of the performance of 102 countries in managing the COVID-19 pandemic according to the Lowy Institute (13 March 2021)	92	101	100

**Table 2:** Correlation among study variables in Chile, Mexico, and Colombia.

	1	2	3	4
1. Beliefs about negative consequences of COVID-19 vaccine	-			
2. Conspiracy beliefs about COVID-19 vaccine	*0.601* ** 0.593 ** 0.551 **	-		
3. Social influence on COVID-19 vaccination intent	−0.418 ** −0.231 ** −0.187 **	−0.581 ** −0.318 ** −0.281 **	*-*	
4. COVID-19 vaccination intent	−0.534 ** −0.508 ** −0.439 **	−0.753 ** −0.652 ** −0.599 **	0.742 ** 0.437 ** 0.440 **	*-*
Mean (SD)	*1.875 (0.924)* **1.761 (0.804)** 2.156 (0.868)	*2.289 (0.866)* **2.308 (0.745)** 2.454 (0.783)	*3.853 (1.330)* **3.920 (1.205)** 3.861 (1.228)	*3.963 (1.234)* **4.167 (1.084)** 3.912 (1.177)

Note. Chilean data are in italics, Mexican data are in bold, and Colombian data are in grey. ** *p* ≤ 0.01.

**Table 3:** Factor loadings and regression coefficients of the model explaining COVID-19 vaccination intent.

Model	χ ^2^	*df*	CFI	TLI	RMSEA(90% CI)	SRMR	Model Comparison	∆CFI	∆RMSEA	Decision
Model 1: Full configural invariance	316.226 **	216	0.995	0.993	0.026 (0.019, 0.032)	0.036	-	-	-	Accept
Model 2: Full metric invariance	364.137 **	230	0.993	0.991	0.029 (0.023, 0.035)	0.038	Model 2 vs. Model 1	−0.002	−0.003	Accept
Model 3: Full scalar invariance	472.256 **	249	0.988	0.986	0.036 (0.031, 0.041)	0.043	Model 3 vs. Model 2	−0.005	−0.007	Accept
Model 4: Full strict invariance	553.248 **	270	0.985	0.984	0.039 (0.034, 0.044)	0.049	Model 4 vs. Model 3	−0.003	0.003	Accept
Model 5: Full structural invariance	633.440 **	300	0.982	0.983	0.040 (0.036, 0.045)	0.052	Model 5 vs. Model 4	−0.003	0.001	Accept

Note. ** *p* < 0.01.

**Table 4:** Factor loadings and regression coefficients of the model explaining COVID-19 vaccination intent.

	Measurement Models Factor Loadings (Standard Error)			Structural Model: COVID-19 Vaccination Intent Standardized Coefficient (Standard Error)		
	Chile	Mexico	Colombia	Chile	Mexico	Colombia
Conspiracy beliefs about COVID-19 vaccine				−0.053 (0.050)	−0.052 (0.050)	−0.054 (0.050)
1. The COVID-19 vaccine will contain a microchip to monitor people	0.796 ** (-)	0.749 ** (-)	0.764 ** (-)			
2. The vaccine against COVID-19 has already been created, but they are withholding it to maintain control of the population	0.790 ** (0.049)	0.742 ** (0.049)	0.758 ** (0.049)			
3. Big Pharma created COVID-19 to benefit from vaccines	0.792 ** (0.053)	0.743 ** (0.053)	0.759 ** (0.053)			
Beliefs about negative consequences of COVID-19 vaccine				−0.591 ** (0.066)	−0.568 ** (0.066)	−0.589 ** (0.066)
1. The COVID-19 vaccine may increase the spread of the virus	0.680 ** (-)	0.616 ** (-)	0.634 ** (-)			
2. I distrust the long-term effectiveness of the COVID-19 vaccine	0.659 ** (0.051)	0.594 ** (0.051)	0.613 ** (0.051)			
3. If I get vaccinated against COVID-19, my chances of contracting the virus increase	0.719 ** (0.038)	0.657 ** (0.038)	0.675 ** (0.038)			
4. The COVID-19 vaccine will cause more complex effects than the virus can have	0.847 ** (0.046)	0.802 ** (0.046)	0.816 ** (0.046)			
5. I think the COVID-19 vaccine has more risks than other vaccines	0.772 ** (0.051)	0.715 ** (0.051)	0.732 ** (0.051)			
6. I am afraid of the possible adverse effects of the COVID-19 vaccine	0.694 ** (0.058)	0.630 ** (0.058)	0.649 ** (0.058)			
Social influence on COVID-19 vaccination intent				0.252 ** (0.023)	0.248 ** (0.023)	0.283 ** (0.023)
R^2^				0.665	0.564	0.575

Note. ** *p* < 0.01.

## Data Availability

Not applicable.

## Supplementary Materials

The following supporting information can be downloaded at: https://www.mdpi.com/article/10.3390/vaccines10071129/s1, Table S1: Sociodemographic characteristics and comparison between groups, Table S2: Standardized regression coefficients and standard errors (in parenthesis) of the model explaining beliefs about negative consequences of COVID-19 vaccine, conspiracy beliefs about COVID-19 vaccine, and social influence on COVID-19 vaccination intent. Table S3: Questions and answer for each item of the study.
