# Supplementary material for: Predictors of COVID-19 Vaccine Intention: Evidence from Chile, Mexico, and Colombia
Source: Vaccines (Basel). 2022 Jul 15;10(7):1129. doi: 10.3390/vaccines10071129 (PMC9318622; doi:10.3390/vaccines10071129)
Supplement: Supplementary file 1 [file vaccines-10-01129-s001.zip › vaccines-1777047-supplementary.pdf]

Supplementary Table S1: Sociodemographic characteristics and comparison between groups.

	Chile	Mexico	Colombia	Difference test
Sex				χ^2 (4) = 6.68; p = .154
Women	731	387	356	
Men	288	176	168	
Age	36.285 (13.77)	29.025 (11.01)	39.395 (17.23)	$F_{(2, 2107)}$ = 80.453, p < .001
Marital status				χ^2 (10) = 3233.399; p < .001
Single	528	366	232	
Married	301	72	180	
Widowed	15	101	13	
Divorced	51	10	13	
Separated	34	4	15	
Cohabiting	94	10	69	
Socioeconomic status				χ^2 (8) = 134.577; p < .001
Lower class	21	12	50	
Lower-middle class	126	79	86	
Middle class	420	320	199	
Upper-middle class	333	137	127	
Upper class	119	15	57	
Educational level				χ^2 (18) = 767.529; p < .001
No formal education	1	3	0	
Complete primary and middle school	3	1	3	
Incomplete high school	6	1	62	
Complete high school	105	10	69	
Incomplete higher education	248	19	61	
Complete higher education	375	118	195	

Incomplete postgraduate degree	62	129	32	
Complete postgraduate degree	222	178	98	
Political orientation				χ^2 (8) = 312.101; p < .001
Far-left	276	97	241	
Center-left	259	80	106	
Center	255	308	161	
Center-right	155	49	0	
Far-right	62	29	0	

Supplementary Table S2: Standardized regression coefficients and standard errors (in parenthesis) of the model explaining beliefs about negative consequences of COVID-19 vaccine, conspiracy beliefs about COVID-19 vaccine, and social influence on COVID-19 vaccination intent.

Socio-structural variables	Beliefs about negative consequences of COVID-19 vaccine			Conspiracy beliefs about COVID-19 vaccine			Social influence on COVID-19 vaccination intent		
	Chile	Mexico	Colombia	Chile	Mexico	Colombia	Chile	Mexico	Colombia
Socioeconomic status	-.127** (.016)	-.110** (.016)	-.122** (.016)	-.150** (.017)	-.128** (.017)	-.198** (.017)	.103** (.031)	.076** (.031)	.122** (.031)
Political orientation	.044 (.013)	.044 (.013)	.036 (.013)	.066** (.015)	.064* (.015)	.053* (.015)	-.039 (.026)	-.033 (.026)	-.028 (.026)
Educational level	-.119** (.012)	-.169** (.010)	-.165** (.010)	-.126** (.012)	-.176** (.012)	-.172** (.012)	.063** (.019)	.076** (.019)	.077** (.019)
Age	.006 (.001)	.006 (.001)	.006 (.001)	.109** (.001)	.102** (.002)	.144** (.001)	-.104** (.012)	-.085** (.002)	-.124** (.002)
R^2		.043			.058			.026	

Note. ** $p \leq .001$, * $p \leq .05$

Supplementary Table S3: Questions and answer for each item of the study.

Scale and item	Response format				
**Conspiracy beliefs about COVID-19 vaccine**	Strongly disagree (1)	Disagree (2)	Neither disagree or agree (3)	Agree (4)	Strongly agree (5)
The COVID-19 vaccine will contain a microchip to monitor people					
The vaccine against COVID-19 has already been created, but they are withholding it to maintain control of the population					
Big Pharma created COVID-19 to benefit from vaccines					
**Beliefs about negative consequences of COVID-19 vaccine**	Strongly disagree (1)	Disagree (2)	Neither disagree or agree (3)	Agree (4)	Strongly agree (5)
The COVID-19 vaccine may increase the spread of the virus					
I distrust the long-term effectiveness of the COVID-19 vaccine					
If I get vaccinated against COVID-19, my chances of contracting the virus increase					
The COVID-19 vaccine will cause more complex effects than the virus can have					
I think the COVID-19 vaccine has more risks than other vaccines					
I am afraid of the possible adverse effects of the COVID-19 vaccine					
**Social influence on COVID-19 vaccination intent**	Strongly disagree (1)	Disagree (2)	Neither disagree or agree (3)	Agree (4)	Strongly agree (5)
I would consider getting vaccinated against COVID-19 if someone close to me does it					
**Vaccination intent against COVID-19**	Not likely (0)	Unlikely (1)	Moderately likely (2)	Very Likely (3)	Extremely likely (4)
How likely are you to that you will be vaccinated against COVID-19?					
